# Effects of Ni Content and Heat Treatment on the Properties, Microstructures, and Precipitates of Cu-0.2 wt% Be-x wt% Ni Alloys

**DOI:** 10.3390/ma17040816

**Published:** 2024-02-08

**Authors:** Yuhan Meng, Bowen Zhang, Jinyun Wang, Zhenyu Hong, Hongliang Zhao, Na Yan, Liang Hu

**Affiliations:** 1 School of Physical Science and Technology, Northwestern Polytechnical University, Xi’an 710072, China; y.h.meng@mail.nwpu.edu.cn (Y.M.); 2017202263@mail.nwpu.edu.cn (B.Z.); wangjunyun@mail.nwpu.edu.cn (J.W.); yanna@nwpu.edu.cn (N.Y.); lhu@nwpu.edu.cn (L.H.); 2 School of Materials Science and Engineering, Zhengzhou University, Zhengzhou 450001, China; zhlwkr@zzu.edu.cn

**Keywords:** Cu-Be alloys, solid solution, aging, electrical conductivity, microhardness, yield strength

## Abstract

Cu-Be alloys exhibit excellent comprehensive performance in electrics, thermotics, and mechanics, and hence, they attract much attention. Among them, low-Be copper alloys are more environmentally friendly and promising. This study explores the effects of different Ni contents and heat treatment parameters on the properties, microstructures, and precipitates of Cu-0.2 wt% Be-x wt% Ni (0 < x < 2.0) alloys. The experimental results demonstrate that the fast cooling rate of cast alloys during solidification contributes to retention of the solute atoms in the copper matrix, which is beneficial for subsequent solid solution treatment. Furthermore, solid solution treatment slightly reduces the electrical conductivities, microhardness values, and compressive yield strengths of Cu-0.2 wt% Be-1.0/1.6 wt% Ni alloys. The optimal solution temperature and time are about 925 ℃ and 60 min, respectively. Aging treatment significantly increases the electrical conductivities, microhardness values, and compressive yield strengths of Cu-0.2 wt% Be-1.0/1.6 wt% Ni alloys. The best aging temperature is around 450 ℃. However, the properties of Cu-0.2 wt%Be-0.4 wt%Ni alloys remain unaffected by solution and aging treatments. Around x = 1.0, Cu-0.2 wt% Be-x wt% Ni alloys possess the best comprehensive properties, which are about 72%IACS of electrical conductivity, 241 HV of microhardness, and 281MPa of compressive yield strength, respectively. TEM and EDS analyses reveal that the precipitate evolution of Cu-0.2 wt% Be-1.0 wt% Ni alloys with aging time is GP zones → γ″ → γ′. Notably, a distinct double-peak age strengthening phenomenon emerges with Cu-0.2 wt% Be-1.0/1.6 wt% Ni alloys. The precipitation of plenty of GP zones at the early stage of aging should account for the first strengthening peak, and the strengthening mechanism transformation of the γ″ or γ′ phase from shear to Orowan should induce the second strengthening peak. This work may help to design new low-Be copper alloys and their preparation processes.

## 1. Introduction

Cu-Be alloys are typical precipitation strengthening alloys with excellent performance in electrics, thermodynamics, mechanics, and chemistry, and hence, they have been broadly applied in the industries of aerospace, electronics, semiconductors, etc. [1,2,3,4]. Investigations to discover the optimal compositions and heat treatment process parameters and to understand the underlying strengthening mechanisms are significant for designing new Cu-Be alloys and developing their preparation processes [5,6,7,8].

To date, most investigations on Cu-Be alloys have been conducted with high-Be copper alloys (1.4–2.0 wt%Be). For example, Rioja and Laughlin reported that the precipitation process of Cu-2%Be alloys was Be equiaxed clusters → GP zones → γ″ → γ′ → γ, as a function of temperature and the quenching method [9]. Yagmur et al. observed the orientation relationship between the copper matrix and the metastable γ′ phase in the Cu-2 wt%Be alloy [10]. Xie et al. presented observations that the precipitation process of the Cu-2%Be alloy was GP zones → γ″ → γ′ → γ, and the main strengthening mechanism was carried out through the bycutting of γ″ and γ′ [11]. Zhang et al. illustrated that the precipitation process of the Cu-1.7 wt%Be alloy was also GP zones → γ″ → γ′ → γ, and the main strengthening mechanism was carried out through the bypassing of γ′ [12]. The hardness, tensile strength, yield strength, and electrical conductivity of the aged Cu-1.7%Be alloy reached 375 HV, 1042 MPa, 778 MPa, and 22.4% IACS, respectively. Chen and Jiang declared that, for the aged Cu-2 wt%Be alloy, the strengthening mechanism of γ″ is carried out through bycutting, while that of γ′ is carried out through bypassing [13]. To sum up, for high-Be copper alloys, the general precipitate evolution during aging treatment is GP zones → γ″ → γ′ → γ, there is only one strengthening peak induced by the mechanism transformation from bycutting to bypassing, and the main strengthening phases are γ″ and/or γ′. High-Be copper alloys have relatively low electrical and thermal conductivity, which proves insufficient.

With the development of modern industries, copper alloys with both high strength and high electrical (or thermal) conductivity are in urgent demand for different applications, such as contact wires over high-speed railways, lead frames under a semiconductor chip, toroidal field coils within a nuclear fusion reactor, and roll shells in a double roller caster [14]. On the other hand, with the development of modern societies, people wish to use more environmentally friendly materials. To our knowledge, low-Be copper alloys are more environmentally friendly with higher electrical conductivity. Therefore, determining how to enhance the strengths of low-Be copper alloys while keeping their high electrical (or thermal) conductivities is very significant.

In the past, Miki and Ogino found that adding Ni into Cu-Be alloys could hasten the precipitation of the γ′ phase and suppress the grain growth during the aging treatment [15]. Peng et al. reported that the peak yield strength and electrical conductivity of Cu-0.34 wt%Be-2.04 wt%Ni alloys were close to 600 MPa and 60% IACS, respectively [16]. Jiang et al. unveiled that the peak hardness, yield strength, and electrical conductivity of Cu-0.4 wt%Be-1.5 wt%Ni alloys were close to 383 HV, 565 MPa, and 47% IACS, respectively [17]. Watanabe et al. declared that the precipitation process of Cu-Be-Ni alloys during aging is also GP zones → γ″ → γ′ → γ [18]. Therefore, adding Ni into low-Be copper alloys can considerably enhance their strengths and, at the same time, keep their high electrical conductivities. The strengthening mechanism of Cu-Be-Ni alloys seems similar to that of high-Be copper alloys.

In this paper, we focus on low-Be copper alloys of Cu-0.2 wt% Be-x wt% Ni (0 < x < 2.0), which are more environmentally friendly, with an electrical conductivity that is higher than ever. The effects of different Ni contents and heat treatment parameters on their properties, microstructures, and precipitates are investigated to determine the optimal Ni content and heat treatment parameters for them. At the same time, we try to understand their underlying strengthening mechanisms during the aging treatment. Performance testers are utilized to test the electrical conductivity, microhardness, and compressive yield strength properties. Metallographic equipment, including an optical microscope (OM), transmission electron microscope (TEM), and energy-dispersive spectroscope (EDS), are employed to analyze the microstructures and precipitates.

## 2. Materials and Methods

Initially, many mixtures containing pure copper (99.99%), nickel (99.99%), and Cu-1.84 wt%Be master alloy were weighed upon the compositions of the target Cu-0.2 wt% Be-x wt% Ni cast alloys (see Supplementary Table S1). Each mixture had a mass of 10 g. Then, they were sealed in vacuum quartz tubes to prevent the escape of the Be element during heating. Next, all of the mixtures were melted and solidified for two cycles in a 10 kW electromagnetic induction (EMI) melting setup to obtain the cast samples. The solidification temperature was recorded by using an infrared temperature detector (IT, METIS M3, Sensortherm, Berlin, Germany), and the average cooling rate was about 30 K/s. After solidification, each cast sample was reweighed to guarantee that the relative mass loss was less than 0.1%. The cast samples had a dimension of 12 mm in diameter and 10 mm in height. After that, some cast samples were remelted and solidified in an electric resistance furnace (ERF) at an extremely low heating and cooling rate (10 K/min) to form the near-equilibrium solidification (NES) samples. Simultaneously, other cast samples were thermally treated at various temperatures for different durations in ERF. Then, the heated samples were quenched in water around 15 °C to obtain solid solution samples. Subsequently, the solution samples were further heated at lower temperatures for various durations in the ERF to produce aged samples.

Thereafter, the electrical conductivity, microhardness, and compressive yield strength of the above samples were, respectively, tested by utilizing a conductivity meter (Sigma 2000B, Sigma-Aldrich, St. Louis, MO, USA), a hardness tester (MacroVicker HV 10, United Testing Systems Canada Ltd., Concord, ON, Canada) with a 50 g load, and a mechanical tester (Instron 5969, Instron, Norwood, MA, USA). The electrical conductivity and microhardness for each sample had been evaluated by averaging 10 and 15 measured values, respectively. The specimens for the compressive yield strength test had a diameter of 3 mm and height of 5.5 mm, and the compression deformation rate during the test was 1% per second.

After performance tests, some selected samples were metallographically analyzed by employing an OM (IMM 5000, PACE Technologies, Tucson, AZ, USA), double Cs corrector TEM (FEI Themis Z, FEI, Inc., Valley City, ND, USA), and EDS (ThermoFisher NS7, Waltham, MA, USA). For optical observation, the samples were etched with a solution of 5 g FeCl_3_ + 20 mL HCl + 100 mL H_2_O. The average grain size of each sample was assessed by randomly selecting 50 OM images of different regions in a sample and measuring the diameters of all grains via commercial software (nano measurement 1.2). For TEM and EDS analyses, the samples were mechanically ground to a thickness of less than 50 µm and further thinned by Ar ions.

## 3. Results and Discussion

### 3.1. Properties of Cast and NES Samples

**Figure 1:**
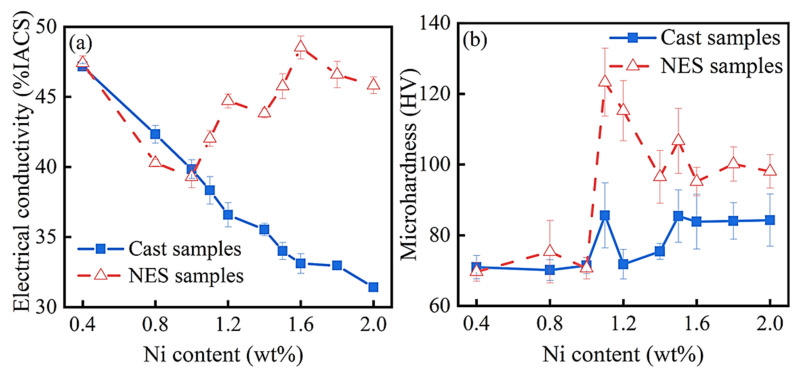
The properties of the cast and NES samples of Cu-0.2 wt% Be-x wt% Ni alloys under different Ni content. (**a**) Electrical conductivity; (**b**) Vickers microhardness.

Figure 1 illustrates the changes in electrical conductivity and Vickers microhardness of the cast and NES samples with the increase in Ni content (0 < x < 2.0). The cast samples were solidified under a cooling rate of about 30 K/s, while the NES samples were solidified under a cooling rate of around 10 K/min. For the cast samples, the electrical conductivity decreases almost linearly from 47%IACS to 31%IACS, and the microhardness value increases slightly and zigzags from 70 HV to 86 HV. The peak microhardness is around 1.0 wt% Ni. For the NES samples, the electrical conductivity first decreases from 47% IACS to 39% IACS, then increases from 39% IACS to 48% IACS, and subsequently decreases again. The turning points occur at 1.0 wt% and 1.6 wt% Ni, respectively. For the same NES samples, the microhardness value initially remains unchanged, then rises sharply from 70 HV to 125 HV, and subsequently decreases again. The peak microhardness value is also around 1.0 wt% Ni.

As suggested in Figure 1 (see also Supplementary Figure S1; refer to electronic Supplementary Materials), the properties and microstructures of the Cu-0.2 wt% Be-x wt% Ni alloys depend on both the Ni content and the cooling rates during solidification. When the Ni content is less than 1.0 wt%, all the solute atoms can be solubilized in the copper matrix for both the fast and slow cooling rates, causing the electrical conductivity to decrease gradually as the Ni content rises. In this case, the solution-strengthening mechanism plays a major role. However, when the Ni content is close to or larger than 1.0 wt%, the properties and microstructures are strongly related to the cooling rate during solidification. Under the fast cooling rate, most solute atoms have insufficient time to remain solubilized in the copper matrix and the solution-strengthening mechanism still plays a major role. Conversely, under the slow cooling rate, most solute atoms have enough time to precipitate from the copper matrix and the precipitation-strengthening mechanism becomes more important. These differences can be understood through the solute trapping effect under a fast cooling rate during solidification [19]. From the perspective of subsequent solid solution treatment, it is thought that a fast cooling rate during the solidification of cast samples is more beneficial. Considering the appearance of peak microhardness around 1.0 wt% Ni in both cases, the Cu-0.2 wt% Be-x wt% Ni cast samples with x = 0.4, 1.0, 1.6 are chosen to be investigated in the following phases of heat treatment.

### 3.2. Properties of Solid Solution Samples

**Figure 2:**
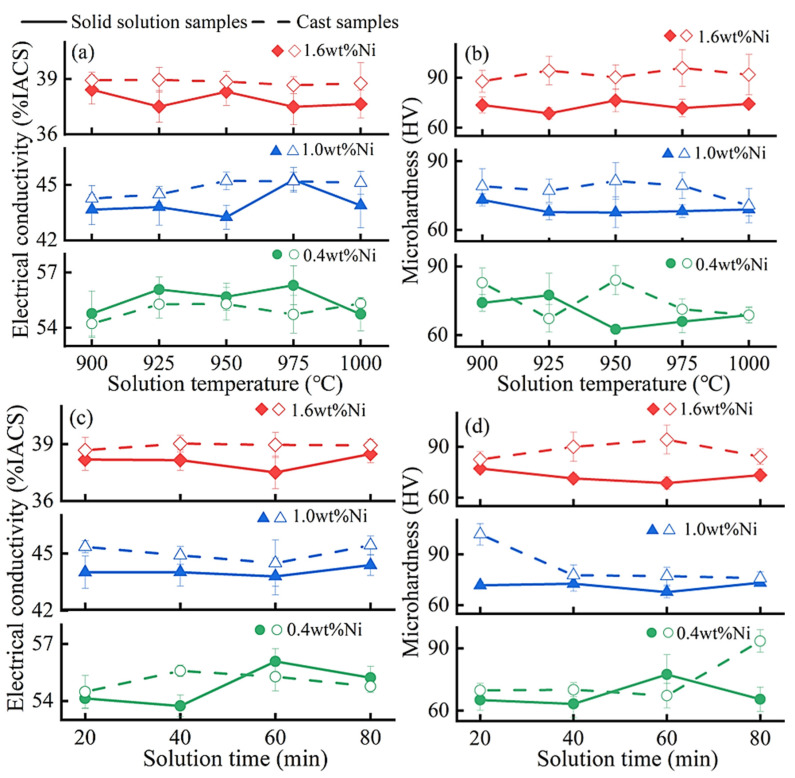
The electrical conductivities (**a**,**c**) and Vickers microhardness (**b**,**d**) of the Cu-0.2 wt%Be-0.4/1.0/1.6 wt%Ni alloys versus the solid solution temperature (**a**,**b**) and time (**c**,**d**). In (**a**,**b**), the solution time is fixed at 60 min. In (**c**,**d**), the solution temperature is fixed at 925 °C. The dashed line signifies the cast samples and the solid line indicates the solid solution samples.

Figure 2 exhibits the effects of different solid solution parameters on the electrical conductivities and Vickers microhardness values of the Cu-0.2 wt% Be-0.4/1.0/1.6 wt% Ni alloys. For the Cu-0.2 wt% Be-0.4 wt% Ni alloy, the electrical conductivity and microhardness change subtly and randomly with the solution temperature and time. However, for Cu-0.2 wt% Be-1.0/1.6 wt% Ni, both electrical conductivities and microhardness values reach their minimum values at an aging temperature range of 925–950 °C and an aging time of 60 min. Through numerous experiments, it has been consistently demonstrated that when the aging parameters are around 925 °C and 60 min, both the electrical conductivity and microhardness are the lowest. 

The purpose of solid solution treatment is to ensure the maximum dissolution of solute elements into the Cu matrix. The solute atoms dissolved in the Cu matrix cause lattice distortion, leading to decreased electrical conductivity. Simultaneously, the dissolution of intermetallic compounds into the matrix results in a reduction in microhardness. The degree of reduction in the electrical conductivity and microhardness reflects the extent of solute atom dissolution in the Cu matrix. Based on the above analysis, under the condition of solid solution treatment, 925 °C for 60 min, the average decline in the electrical conductivity and microhardness is the most significant. Therefore, the optimal solid solution parameters for the Cu-0.2 wt% Be-1.0/1.6 wt% Ni alloys are around 925 °C and 60 min.

Simultaneously, the experimental results above indicate that when the Ni content is very low (around 0.4 wt%), no precipitation occurs from the cast samples, and as a result, the solid solution treatment has little effect on the Cu-0.2 wt% Be-0.4 wt% Ni alloys. But with a higher Ni content, as that in the Cu-0.2 wt% Be-1.0/1.6 wt% Ni alloys, precipitation from the cast samples takes place. In this case, the precipitates redissolve into the copper matrix during the solid solution treatment, leading to the lattice distortion of the copper matrix and the reductions in electrical conductivity and microhardness [20].

Additionally (see Supplementary Figure S2), many twin crystals are found in the solid solution-treated samples, which is due to the low stacking fault energy of the copper matrix. The average grain sizes in the solid solution Cu-0.2 wt% Be-0.4/1.0/1.6 wt% Ni alloys treated by 925 °C for 20 min are 73.8, 66.6, and 52.5 μm, respectively, increasing to 74, 67, and 53 μm after 60 min, signifying the role of Ni in grain refinement. 

### 3.3. Properties of Aged Samples

**Figure 3:**
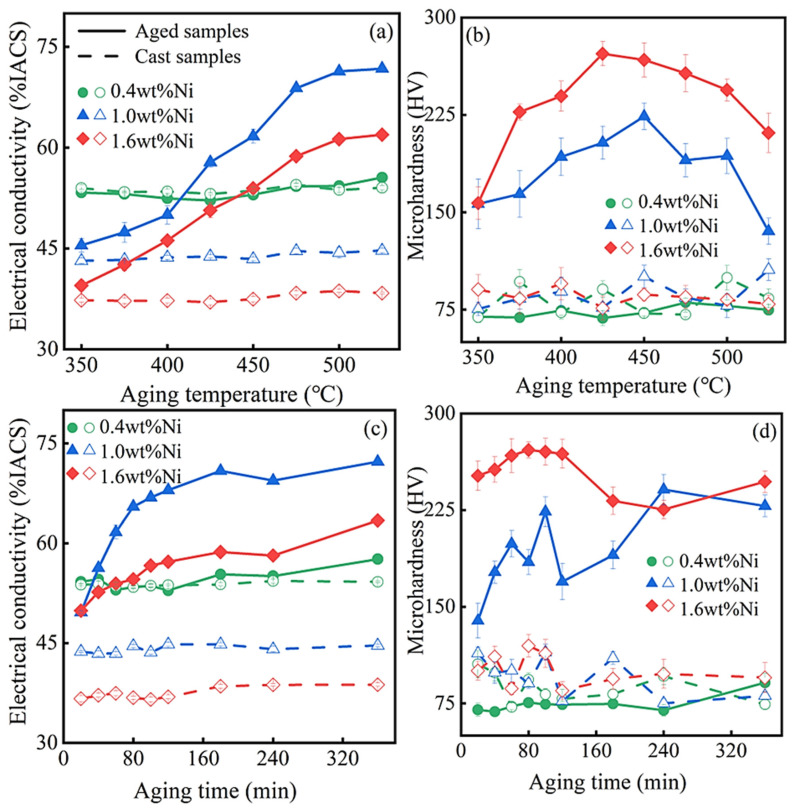
The electrical conductivities (**a**,**c**) and Vickers microhardness (**b**,**d**) of the Cu-0.2 wt% Be-0.4/1.0/1.6 wt% Ni alloys versus the aging temperature (**a**,**b**) and time (**c**,**d**). In (**a**,**b**), the aging time is 60 min. In (**c**,**d**), the aging temperature is 450 °C. The dashed line signifies the cast samples and the solid line indicates the aged samples.

Figure 3 illustrates the effects of various aging parameters on the electrical conductivity and Vickers microhardness of the Cu-0.2 wt% Be-0.4/1.0/1.6 wt% Ni alloys. At first, the aging time is fixed at 60 min, and the aging temperature is varied to find the alloys’ best parameter. From Figure 3a, it can be observed that, for the Cu-0.2 wt% Be-1.0/1.6 wt% Ni alloys, the electrical conductivities of the two alloys continuously increase with the increase in aging temperature from 350 to 525 °C. At 525 °C, they reach their maximum values, 71.5 and 61.9%IACS, respectively. Under the same aging conditions (Figure 3b), the microhardness values of the Cu-0.2 wt% Be-1.0 wt%/1.6 wt% Ni alloys initially rise, and then decrease with the increase in aging temperature. Around 450 °C, they reach their maximum values, 224 and 272 HV, respectively. However, from Figure 3a,b, no significant improvements in electrical conductivity and microhardness have been observed for the Cu-0.2 wt% Be-0.4 wt% Ni alloy.

Based on the above results, the aging temperature is fixed at 450 °C and the aging time is varied to find its best parameter. From Figure 3c, we can see that the electrical conductivities of the Cu-0.2 wt% Be-1.0 wt%/1.6 wt% Ni alloys climb continuously with the increase in aging time from 20 to 360 min. At 360 min, they reach their maximum values, 72.2 and 63.4% IACS, respectively. Under the same aging conditions (Figure 3d), the microhardness values of the Cu-0.2 wt% Be-1.0/1.6 wt% Ni alloys increase initially followed by a subsequent decrease, and then undergo another increase and decrease with the increase in aging time, demonstrating a clear double-peak aging strengthening phenomenon. For the Cu-0.2 wt% Be-1.0 wt% Ni alloy, the first and second microhardness peaks occur around 60 and 240 min, measuring 224 and 241 HV, respectively. For the Cu-0.2 wt% Be-1.6 wt% Ni alloy, the first and second microhardness peaks appear around 80 and 360 min, measuring 272 and 247 HV, respectively. However, from Figure 3c,d, no significant improvements in electrical conductivity and microhardness have been observed for the Cu-0.2 wt% Be-0.4 wt% Ni alloy. 

The experimental results above suggest that when the Ni content in the Cu-0.2 wt% Be-x wt% Ni alloys is very low (around 0.4 wt%), aging treatment also has little influence on their properties. Otherwise, higher Ni content leads to notable enhancements in electrical conductivity and microhardness through aging treatment. In this case, there exists a distinct double-peak age strengthening phenomenon [21]. Overall, when considering the electrical conductivity and microhardness comprehensively, the optimal properties of the Cu-0.2 wt% Be-x wt% Ni alloys can be attained at approximately x = 1.0, where the conductivity and microhardness are about 70% IACS and 241 HV, respectively.

**Figure 4:**
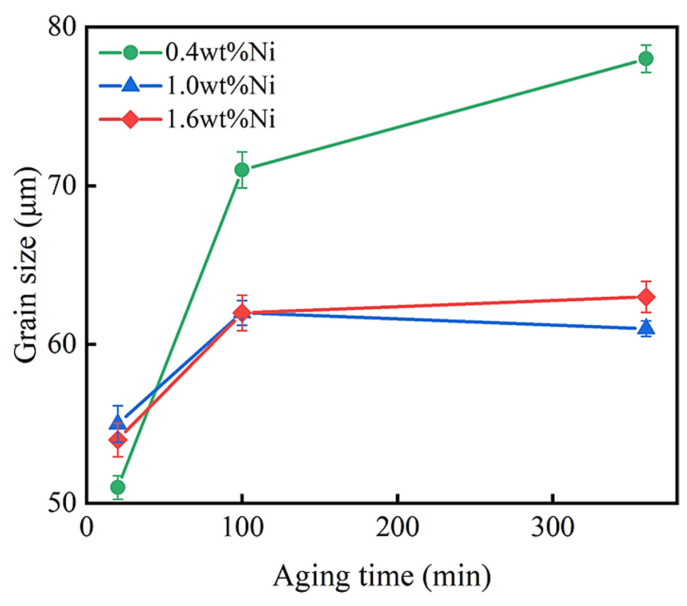
The average grain sizes of Cu-0.2 wt% Be-x wt% Ni alloys under different aging times.

Figure 4 presents the average grain sizes of the Cu-0.2 wt% Be-0.4/1.0/1.6 wt% Ni alloys with the aging time increasing from 20 to 360 min at a fixed aging temperature of 450 ℃. The sizes increase from 51, 56, and 55 μm at 20 min to 79, 63, and 62 μm at 360 min, respectively. It was found that the size first increased slightly and then remained unchanged with the increase in aging time. These data also suggest that the increase in Ni content can effectively inhibit the grain growth during aging treatment. Additionally, the microstructures of the aged samples mainly consist of twin crystals (see Supplementary Figure S3), similar to that in the solid solution samples.

### 3.4. Compressive Yield Strengths of Different Samples

**Figure 5:**
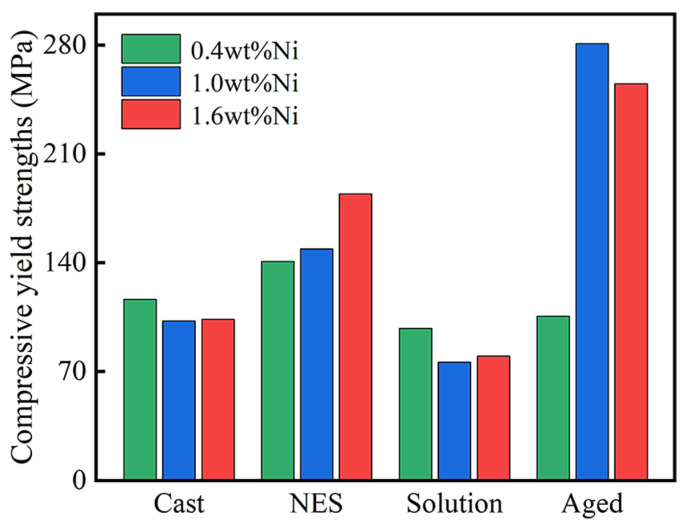
The compressive yield strengths of the cast, NES, solution, and aged samples of Cu-0.2 wt% Be-x wt% Ni alloys under different Ni content.

Figure 5 exhibits the compressive yield strengths of the cast, NES, solution (at 925 °C for 60 min), and aged (at 450 °C for 100 min) samples of the Cu-0.2 wt% Be-0.4/1.0/1.6 wt% Ni alloys. For the Cu-0.2 wt% Be-0.4 wt% Ni alloy, the compressive yield strengths of the solution and aged samples are smaller than those of the cast and NES samples, indicating that when the Ni content is very low, heat treatment or precipitation strengthening has a negligible effect on it. For the Cu-0.2 wt% Be-1.0/1.6 wt% Ni alloys, aging treatment results in the great enhancement of compressive yield strengths, and their highest values are 281 MPa and 255 MPa, respectively. Therefore, from the perspective of compressive yield strength, the optimal Ni content in the Cu-0.2 wt% Be alloy is around 1.0 wt%, which is consistent with the above results regarding the comprehensive properties of electrical conductivity and microhardness [22].

### 3.5. Precipitate Evolution

**Figure 6:**
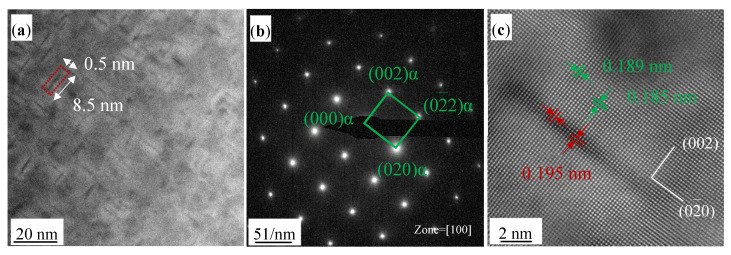
TEM images of the Cu-0.2 wt% Be-1.0 wt% Ni alloy aged at 450 °C for 20 min. (**a**) HAADF image; (**b**) corresponding SAED pattern; (**c**) atomic image of precipitates.

**Figure 7:**
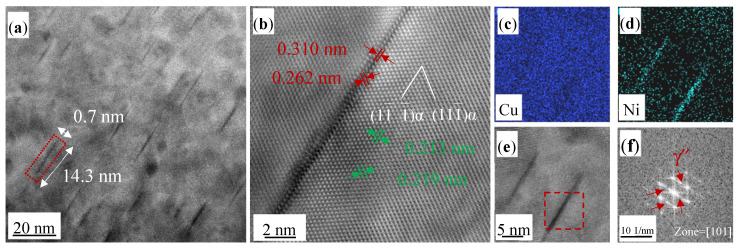
TEM and EDS images of the Cu-0.2 wt% Be-1.0 wt% Ni alloy aged at 450 ℃ for 100 min. (**a**) HADDF image; (**b**) atomic image of precipitates; (**c**,**d**) EDS element map of Cu and Ni; (**e**,**f**) HRTEM image and corresponding FFT patterns of precipitates.

**Figure 8:**
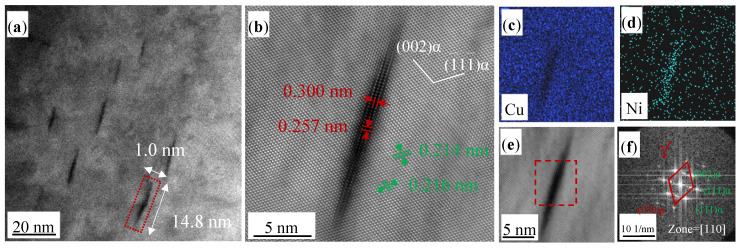
TEM and EDS images of the Cu-0.2 wt% Be-1.0 wt% Ni alloy aged at 450 °C for 360 min. (**a**) HAADF image; (**b**) atomic image of precipitates; (**c**,**d**) EDS element map of Cu and Ni; (**e**,**f**) HRTEM image and corresponding FFT patterns of precipitates.

Figure 6, Figure 7 and Figure 8 provide TEM images of the Cu-0.2 wt% Be-1.0 wt% Ni alloy aged at 450 °C for 20, 100, and 360 min and illuminated at the electron beam direction parallel to [100]_α_, [101]_α_, and [110]_α_, respectively. Figure 6a is the high-angle annular dark field (HAADF) image, where a large number of disk-like precipitates are observed in the matrix. The average diameter and thickness of the precipitates was 8.5 nm and 0.5 nm, respectively. These nanoscale precipitates are arranged vertically alongside each other. As suggested by the corresponding selected area electron diffraction (SAED) pattern in Figure 6b, only one set of regular spots corresponding to the α(Cu) phase can be recognized, although there are some weak streaks visible between the diffraction spots [13]. The atomic image in Figure 6c demonstrates that the crystal structure of the precipitates coincides with that (FCC) of the α(Cu) matrix, and there are noticeable strain fields around the precipitates, which are caused by coherent deformation. Overall, after aging at 450 °C for 20 min, the precipitates in the Cu-0.2 wt% Be-1.0 wt% Ni alloy are mainly in the form of GP zones, which are Be atomic layers stacked parallel to the {100} plane of the α(Cu) matrix.

Figure 7a,b present the HAADF and atomic images of the Cu-0.2 wt% Be-1.0 wt% Ni alloy aged at 450 °C for 100 min, respectively. It can be observed that the disk-like precipitates have an average diameter of 14.3 nm and thickness of 0.7 nm. These dimensions are larger than those for the 20 min treatment case, shown in Figure 6. They still maintain a coherent relationship with the α(Cu) matrix. The EDS maps in Figure 7c,d reveal that the precipitates are Ni-rich. To further analyze the precipitate structure, the corresponding fast Fourier transform (FFT) analysis of the enclosed square region in Figure 7e is presented in Figure 7f. This FFT pattern indicates that the precipitates are γ″ phase, characterized by a body-centered tetragonal (BCT) structure (a = b = 0.262 nm and c = 0.310 nm).

As exhibited in Figure 8a,b, the precipitates grow further to an average diameter of 14.5 nm and thickness of 1.0 nm at 450 °C for 360 min. They maintain a semi-coherent relationship with the α(Cu) matrix. The EDS maps in Figure 8c,d demonstrate a further enrichment of Ni atoms within the precipitates. In the TEM test, elements with higher atomic numbers appear brighter in the HADDF image. Given that Ni has an atomic number of 28 and Cu has an atomic number of 29, the Ni-rich needle precipitation in Figure 6a, Figure 7a and Figure 8a appears darker compared to the surrounding Cu matrix. The results align with the EDS element maps of Cu and Ni in Figure 7c,d and Figure 8c,d. Additionally, direct observation of light Be atoms via TEM remains difficult. The FFT analysis of the square region in Figure 8e was conducted to determine the precipitate structure, as shown in Figure 8f. It implies that the precipitates are γ′ phase, characterized by a BCT structure (a = b = 0.257 nm and c = 0.300 nm).

To summarize, the precipitate evolution in the Cu-0.2 wt% Be-1.0 wt% Ni alloy during aging treatment is GP zones → γ″ → γ′, in agreement with previous studies [18]. The equilibrium phase, γ, is formed with the further increase in aging time [16].

### 3.6. Discussions of Strengthening Mechanism 

As can be seen in the above results, it is clear that precipitation strengthening, other than solution strengthening, plays a major role in strengthening the Cu-0.2 wt% Be-x wt% Ni alloys when x is close to or larger than 1.0. However, there exists a double-peak age strengthening phenomenon with the Cu-0.2 wt% Be-1.0/1.6 wt% Ni alloys. This phenomenon has not been found before in such Cu-Be-based systems. 

According to Section 3.5, we consider that the traditional strengthening theory can explain the second strengthening peak [23]. That is, the transformation of the strengthening mechanism from shear (bycutting) to Orowan (bypassing) with the growth of precipitates can induce the appearance of a strengthening peak [24,25]. For the Cu-0.2 wt% Be-1.0/1.6 wt% Ni alloys, when the aging time is more than 100 min, the evolution of the coherent γ″ phase to semi-coherent γ′ phase, and further to inherent γ phase, can produce such a transformation. However, this traditional strengthening theory cannot explain the appearance of the other strengthening peak at the early stage of aging.

Regarding the first strengthening peak, we speculate that it is related to the number of GP zones. Although, compared with a γ″ or γ′ precipitate, a single GP zone may play a weaker role in strengthening the matrix [26], the number of GP zones at the beginning stage of aging may be significantly larger than that of the γ″ and γ′ precipitates at the following stage of aging. So, at the beginning stage of aging, the strength can increase sharply with the precipitation of a lot of very small GP zones, and as aging proceeds, it has to decrease to a certain degree due to the rapid transition of GP zones. Thus, the first strengthening peak appears.

Additionally, the occurrences of lots of twin crystals due to heat treatment and grain refinement due to Ni addition must increase the effect of grain boundary strengthening [27,28]. However, because of the large grain sizes (>50 μm), their contributions to the total strength are limited.

## 4. Conclusions

This work investigates the effects of Ni content and heat treatment parameters on the properties, microstructures, and precipitates of Cu-0.2 wt% Be-x wt% Ni (0 < x < 2.0) alloys. The main conclusions are drawn as follows:

1. For cast alloys, a fast cooling rate during solidification can retain more solute atoms in the α(Cu) matrix, which is beneficial for subsequent solid solution treatment.
2. The solid solution treatment has a negligible effect on the properties of Cu-0.2 wt% Be-x wt% Ni alloys when x is small (around 0.4). Otherwise, both the electrical conductivity and microhardness decrease slightly with solid solution treatment. The optimal solution parameters are around 925 °C and 60 min.
3. The aging treatment also has a negligible effect on the properties of Cu-0.2 wt% Be-x wt% Ni alloys when x is small (around 0.4). Otherwise, aging treatment can lead to a continuous increase in electrical conductivity and a double-peak age strengthening phenomenon. Around x = 1.0, the best comprehensive properties of electrical conductivity, microhardness, and compressive yield strength can be obtained, measuring about 72% IACS, 241 HV, and 281 MPa, respectively. The optimal aging parameters for the Cu-0.2 wt% Be-1.0 wt% Ni alloy are 450 °C and 60 or 240 min.
4. With the increase in aging time, the precipitate evolution in the Cu-0.2 wt% Be-1.0 wt% Ni alloy is GP zones → γ″ → γ′. The precipitation of plenty of GP zones accounts for the appearance of the first strengthening peak. The strengthening mechanism transformation from shear to Orowan of the γ″ or γ′ phase explains the appearance of the second strengthening peak.
5. Additionally, lots of twin crystals of the α(Cu) matrix can be produced by solid solution or aging treatments, and adding adequate Ni can effectively prevent the matrix grains from growing during aging treatment.

## Data Availability

The data that support the findings of this study are available from the corresponding author upon reasonable request.

## Supplementary Materials

The following supporting information can be downloaded at: https://www.mdpi.com/article/10.3390/ma17040816/s1, Table S1: Compositions of the three main research alloys; Figure S1: The microstructures of (a,c,e) cast samples and (b,d,f) NES samples of Cu-0.2 wt% Be-x wt% Ni alloys. (a,b) 0.4 wt% Ni; (c,d) 1.0 wt% Ni; (e,f) 1.6 wt% Ni; Figure S2: The microstructures of solid solution samples of Cu-0.2 wt% Be-x wt% Ni alloys at the solid solution temperature 925 °C and time 60 min. (a) 0.4 wt% Ni; (b) 1.0 wt% Ni; (c) 1.6 wt% Ni; Figure S3: The microstructures of aged samples of Cu-0.2 wt% Be-x wt% Ni alloys at the aging temperature 450 °C and time 100 min. (a) 0.4 wt% Ni; (b) 1.0 wt% Ni; (c) 1.6 wt% Ni; Figure S4: Relationship between compression deformation and loading force.
