# Supplementary material for: Effects of Ni Content and Heat Treatment on the Properties, Microstructures, and Precipitates of Cu-0.2 wt% Be-x wt% Ni Alloys
Source: Materials (Basel). 2024 Feb 8;17(4):816. doi: 10.3390/ma17040816 (PMC10890441; doi:10.3390/ma17040816)
Supplement: Supplementary file 1 [file materials-17-00816-s001.zip › materials-2819089-supplementary.pdf]

Effects of Ni content and heat treatment on the properties, microstructures, and precipitates of Cu-0.2wt%Be-xwt%Ni alloys

Supplementary Materials

Table S1: Compositions of the three main research alloys.

Content	1	2	3
Weight Percentage (wt%)	Cu-0.2wt%Be-0.4wt%Ni	Cu-0.2wt%Be-1.0wt%Ni	Cu-0.2wt%Be-1.6wt%Ni
Atomic percentage (at%)	Cu-0.03at%Be-0.37at%Ni	Cu-0.03at%Be-0.92at%Ni	Cu-0.03at%Be-1.48at%Ni

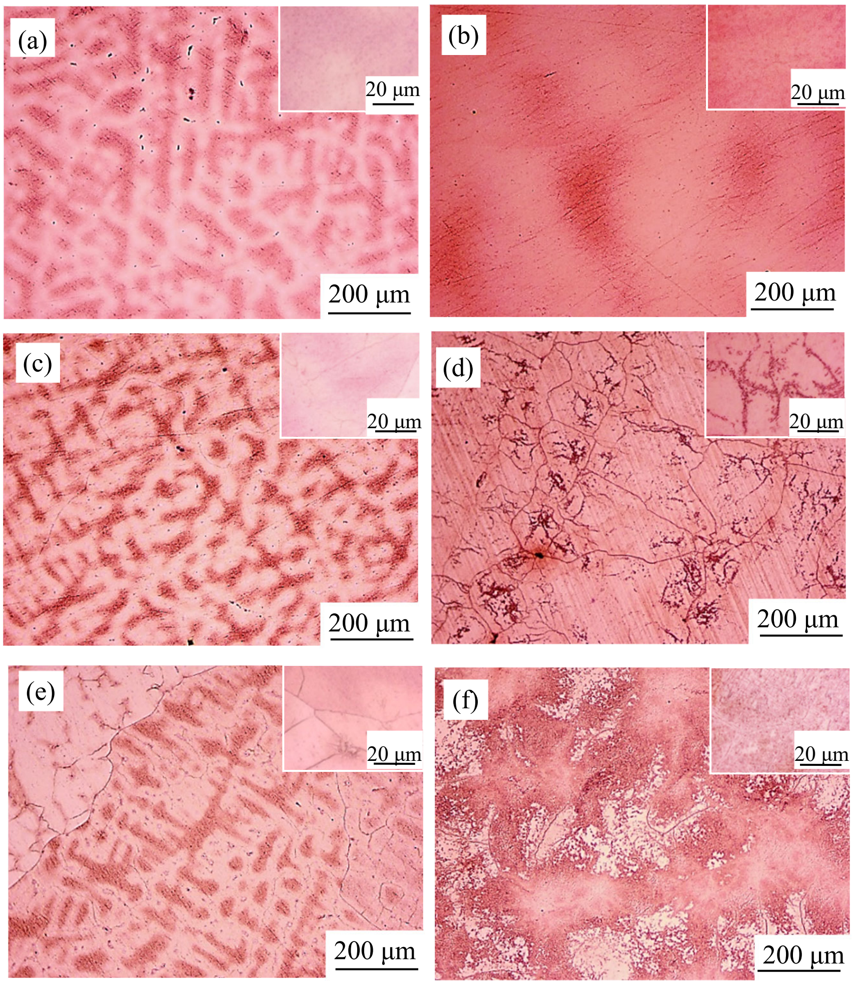

Figure S1: The microstructures of (a,c,e) cast samples and (b,d,f) NES samples of Cu-0.2wt%Be-xwt%Ni alloys. (a,b) 0.4wt%Ni; (c,d) 1.0wt%Ni; (e,f) 1.6wt%Ni.

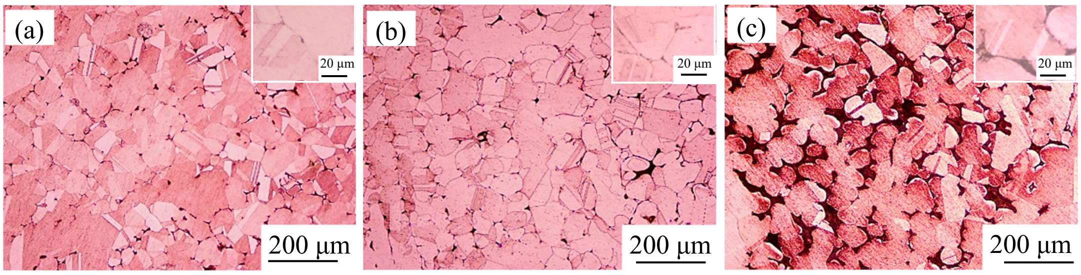

Figure S2: The microstructures of solid solution samples of Cu-0.2wt%Be-xwt%Ni alloys at the solid solution temperature 925℃ and time 60 minutes. (a) 0.4wt%Ni; (b) 1.0wt%Ni; (c) 1.6wt%Ni.

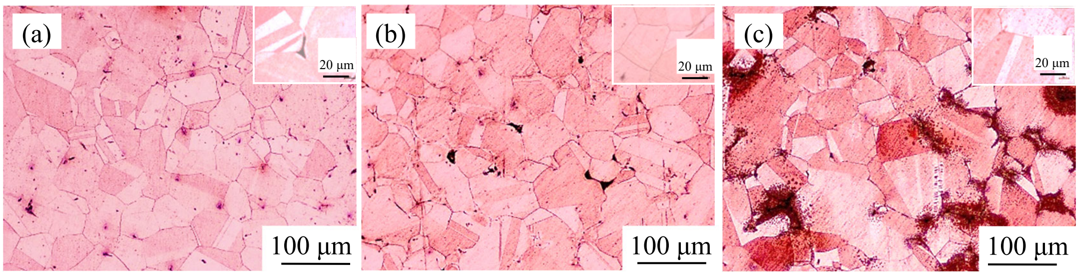

Figure S3: The microstructures of aged samples of Cu-0.2wt%Be-xwt%Ni alloys at the aging temperature 450℃ and time 100 minutes. (a) 0.4wt%Ni; (b) 1.0wt%Ni; (c) 1.6wt%Ni.

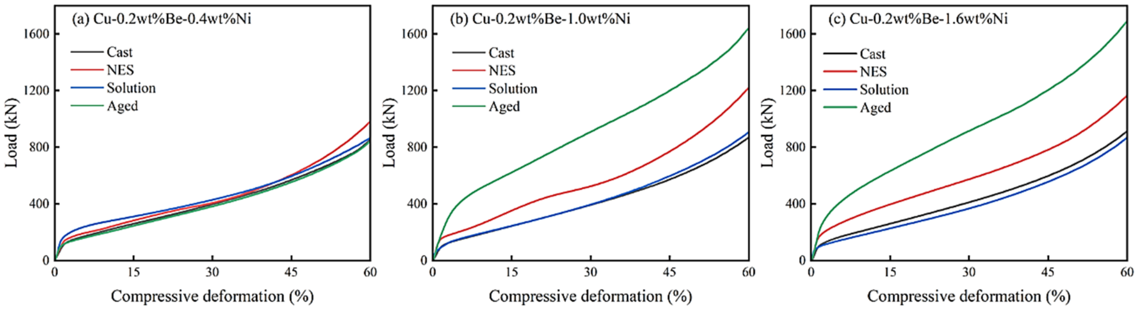

Figure S4: Relationship between compression deformation and loading force.
